# Mobilizable genomic islands, different strategies for the dissemination of multidrug resistance and other adaptive traits

**DOI:** 10.1080/2159256X.2017.1304193

**Published:** 2017-04-12

**Authors:** Nicolas Carraro, Nicolas Rivard, Vincent Burrus, Daniela Ceccarelli

**Affiliations:** a Laboratory of Bacterial Molecular Genetics, Département de Biologie, Faculté des Sciences, Université de Sherbrooke, Sherbrooke, Québec, Canada; b Department of Fundamental Microbiology, University of Lausanne, Lausanne, Switzerland; c Department of Bacteriology and Epidemiology, Wageningen Bioveterinary Research, Lelystad, the Netherlands

**Keywords:** A/C, antibiotic resistance, genomic island, integrative conjugative element, mobilization, plasmid

## Abstract

Mobile genetic elements are near ubiquitous DNA segments that revealed a surprising variety of strategies for their propagation among prokaryotes and between eukaryotes. In bacteria, conjugative elements were shown to be key drivers of evolution and adaptation by efficiently disseminating genes involved in pathogenicity, symbiosis, metabolic pathways, and antibiotic resistance. Conjugative plasmids of the incompatibility groups A and C (A/C) are important vehicles for the dissemination of antibiotic resistance and the consequent global emergence and spread of multi-resistant pathogenic bacteria. Beyond their own mobility, A/C plasmids were also shown to drive the mobility of unrelated non-autonomous mobilizable genomic islands, which may also confer further advantageous traits. In this commentary, we summarize the current knowledge on different classes of A/C-dependent mobilizable genomic islands and we discuss other DNA hitchhikers and their implication in bacterial evolution. Furthermore, we glimpse at the complex genetic network linking autonomous and non-autonomous mobile genetic elements, and at the associated flow of genetic information between bacteria.

Bacterial genomes are dynamic entities subjected to a constant flow of loss and gain of genetic material.^1^ Gene acquisition can provide bacterial hosts with adaptive traits and is likely to confer a selective advantage in particular conditions.^2^ Self-transmissible mobile genetic elements (MGEs) such as prophages and conjugative elements were shown to be an immense resource for genome evolution and bacterial adaptation.^1-3^

Genomic islands (GIs) also largely participate in bacterial genome diversification. Many GIs were identified thanks to the adaptive traits they encoded and named accordingly, e.g. pathogenicity islands or symbiosis islands. GIs are chromosomal DNA segments typically present in subsets of closely related strains, one of the hallmarks of acquisition by horizontal gene transfer. However, the mechanism of acquisition and dissemination of GIs has remained a conundrum for a long time, often due to the lack of obvious mobility–related genes.^4^ Recent studies have refined our understanding of the biology and mobility mechanisms of several of mobilizable GIs (MGIs) families including satellite prophages and GIs mobilized by conjugative elements.^5-7^ Mobility of MGIs involves self-transmissible MGEs that provide them with functions they lack to catalyze their dissemination. Availability of thousands of bacterial genome sequences associated with low-cost, high-throughput modern molecular methods unraveled an even greater diversity of GIs.

We recently reported the discovery of MGI*Vch*Hai6, a new mobile resistance island in *Vibrio cholerae*, that is mobilizable by A/C conjugative plasmids.^8^ Here, we compare the possible mechanisms of activation and mobilization of MGI*Vch*Hai6 with two other A/C-dependent mobilizable GIs as well as a family of GIs mobilized by integrative and conjugative elements (ICEs) of the SXT/R391 family.

## Regulation of transfer of A/C plasmids

Plasmids of the incompatibility groups A and C (A/C) are large (> 110 kb) double-stranded molecules that efficiently disseminate by conjugation.^9^ A/C plasmids drive the spread of multiple antibiotic resistances including last-resort antimicrobial compounds such as carbapenems.^10,11^

Recent studies demonstrated that control of A/C plasmids mobility is reminiscent of the FlhCD-dependent activation of flagellar motility in *Escherichia coli* and related motile bacteria.^12,13^ Nevertheless, the A/C regulatory circuitry is a unique system with specific early molecular actors and plasmid-borne target genes. Two repressors named Acr1 and Acr2 (A/C repressors 1 and 2) repress the constitutive transcription of *acr1* from *P_acr1_*.^13,14^ Upon inducing conditions that remain to be identified, repression of *P_acr1_* is alleviated, allowing not only the transcription of *acr1* but also that of all four downstream genes. Two of these genes, *acaC* and *acaD*, were shown to code for subunits of the FlhCD-like master activator of A/C plasmids AcaCD (A/C activator, subunits C and D).^13^ Thorough investigation revealed that AcaCD targets 18 A/C-borne promoter regions, thereby activating the transcription of genes and operons responsible for conjugative transfer. Surprisingly, genes coding for predicted or demonstrated functions account for a fraction of AcaCD-activated genes as nearly two thirds of these genes code for proteins of unknown function.^13,15^ Future investigation of this large *terra incognita* is necessary to fully understand the biology of A/C plasmids.

## A/C-dependent mobilization of mobilizable genomic islands

Recent work conducted by our group uncovered the extended role of AcaCD.^12^ Besides A/C-borne sequences, AcaCD was shown to recognize chromosomal loci that belong to A/C-unrelated MGIs.^13^

To date, three unrelated families of A/C-dependent MGIs were identified and named after the prototypical elements that were experimentally characterized: MGI*Vch*Hai6 of *V. cholerae*, MGI*Vmi*1 of *V. mimicus* and *Salmonella* genomic island 1 (SGI1).^8,13,16^ Each family encompasses several members sharing a conserved core sequence. Different members of the same family contain distinct insertion of variable DNA coding for adaptive traits or proteins of unknown function.^8,15,17-19^ While the precise molecular mechanism leading to intercellular mobility of these elements remains to be deciphered, accumulation of evidence suggests the pivotal role of genes under the control of AcaCD.

**Figure 1.**
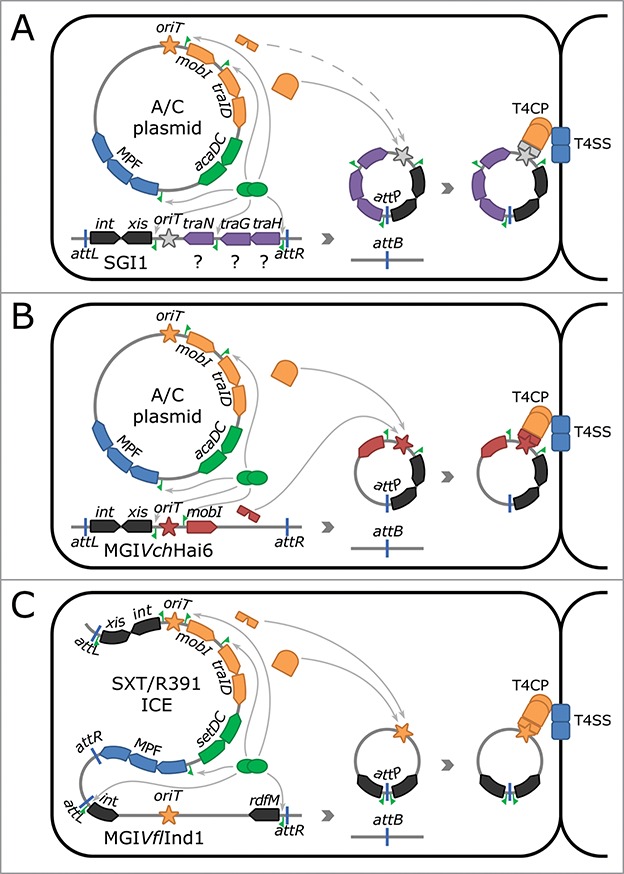
Proposed mobilization mechanisms for three known families of mobilizable genomic islands. (A) Mobilization of SGI1 and related elements by A/C plasmids. (B) Mobilization of MGI*Vch*Hai6 and related elements by A/C plasmids. (C) Mobilization of MGI*Vfl*Ind1 and related elements by SXT/R391 ICEs. Different items are portrayed as follows: arrowed boxes, genes; green pennants, AcaCD/SetCD binding sites; blue strokes, left (*attL*) and right (*attR*) junctions as well as chromosomal (*attB*) and circular form (*attP*) attachment sites; MPF, genes involved in mating pore formation. *oriT*_SGI1_ along with helper plasmid's MobI are depicted in gray and with a dotted line to take into account their highly speculative nature. SGI1 and MGI*Vch*Hai6 are integrated into the 3′ end of *trmE*, whereas SXT/R391 ICEs are integrated into the 5′ end of *prfC*, and MGI*Vfl*Ind1 into the 3′ end of *yicC*.

Based on these observations and the presence of conserved features, we propose two distinct models of mobilization, one for SGI1 and relatives, and the other for MGI*Vch*Hai6/MGI*Vmi*1-like elements (Fig. 1).

## SGI1: First evidence of A/C-dependent MGI

A/C were firstly shown to specifically mobilize GIs in 2005 with the characterization of SGI1 and by extent the large family of SGI1 elements.^13,16,20^ SGI1 and its siblings are recognized as major determinants of multidrug resistance in *Salmonella enterica* and *Proteus mirabilis*.^18^ The integron In104 of SGI1 elements confers multidrug resistance.^18,19^

Five AcaCD binding sites were discovered in SGI1, allowing to posit on the underlying mechanism allowing SGI1 mobilization (Fig. 1A).^13,21,22^ SGI1 carries *int*, a constitutively expressed gene coding for the site-specific integrase that catalyzes the integration of SGI1 into the 3′ end of *trmE* in the chromosome of its host.^16,21,23,24^ Upon arrival of an A/C plasmid in the cell, associated synthesis of AcaCD triggers the transcription of *xis*, a gene coding for a recombination directionality factor (RDF).^16,21^ Xis would displace the integrase-mediated recombination reaction toward excision of SGI1 as a plasmid-like molecule that is the substrate for conjugative transfer (Fig. 1A). To date, no origin of transfer (*oriT*), the *cis*-acting locus where DNA transfer is initiated, has been identified on SGI1. Furthermore, A/C- and SGI1-encoded factors that process this putative *oriT* and enable docking of SGI1 DNA to the mating pore for transfer to the recipient cell remain to be characterized. Once in the recipient, SGI1 is thought to be independent of the helper plasmid in regards to its site-specific integration due to the constitutive expression of *int*.^21^

## Members of the MGI*Vch*Hai6 family are mobilizable by A/C plasmids

MGI*Vch*Hai6 is the prototypical member of a new family of MGIs involved in the dissemination of multidrug resistance.^8^ This 47-kb element was identified in a non-O1/non-O139 *V. cholerae* clinical isolate recovered from a cholera patient in Haiti in 2010.^25^ Like SGI1, MGI*Vch*Hai6 is integrated into the 3′ end of *trmE*. It also carries a distinct integron, In36A1, conferring resistance to β-lactams, florfenicol/chloramphenicol, streptomycin/spectinomycin, sulfamethoxazole and trimethoprim (co-trimoxazole), and tetracycline. MGI*Vch*Hai6 also likely confers resistance to bacteriophage infection and mercury, as it bears a type I restriction-modification system and Tn*6310*. MGI*Vch*Hai6-like elements are globally distributed in environmental and clinical *V. cholerae* isolates recovered from 1977 to 2010. All members of this family of MGIs share a ∼8-kb conserved core that likely ensures essential maintenance and transfer functions. Mobility of MGI*Vch*Hai6 was shown to be strictly dependent on the presence of an A/C plasmid.

Despite its different size and gene content, MGI*Vch*Hai6 shares several features with MGI*Vmi*1, an element integrated into the 3′ end of *yicC*.^8,13,15^ In both MGI*Vch*Hai6 and MGI*Vmi*1, AcaCD was shown to drive the transcription of the RDF gene *xis* and of a gene coding for a MobI-like protein (Fig. 1B). MobI is required for transfer of integrative and conjugative elements (ICEs) of the SXT/R391 family and A/C plasmids.^26,27^ In SXT/R391 ICEs and A/C plasmids, *oriT* is located in a large intergenic region upstream of *mobI*.^26,27^ By analogy, we predict that *oriT* of MGI*Vch*Hai6/MGI*Vmi*1 elements (*oriT*_MGI_) is located in the large intergenic region upstream of *mobI*_MGI_ (Fig. 1B).

A model for MGI*Vch*Hai6/MGI*Vmi*1 lifecycle infers that these elements remain quiescent in their integrated chromosomal state. Based on work done on other MGIs, stable integration of MGI*Vch*Hai6/MGI*Vmi*1 elements is likely enabled by constitutive expression of *int*.^21,28^ Like for SGI1, entry of an A/C plasmid that expresses AcaCD triggers the synthesis of Xis that, in concert with the integrase, mediates the excision of the MGI (Fig. 1B). AcaCD also activates the synthesis of MobI_MGI_ that is thought to recognize and bind to *oriT*_MGI_.^26,27^ MobI_MGI_ would act as an adaptor protein that recruits and assembles the A/C plasmid encoded DNA-processing machinery, called relaxosome, within which the relaxase TraI initiates conjugative transfer through the A/C-encoded mating pore (Fig. 1B). The MGI is then assumed to be able to site-specifically integrate into the genome of the recipient cell regardless of the presence of the A/C plasmid, as expression of the integrase is constitutive.

## MGIs come in many flavors

For several decades, studies of members of well-known families of self-transmissible MGEs eclipsed the discovery of new types of mobile elements. New evidence suggests that MGIs could be more abundant conjugative entities than expected, presaging an unforeseen significant ecological role.^5,29^ So far, their influence has been overlooked likely because of difficulties identifying them and/or identifying their cognate helper element(s).

Early milestones into the discovery of MGIs include the characterization of CTn-dependent non-replicating *Bacteroides* units (NBUs), phage-mobilizable *Staphylococcus aureus* pathogenicity islands (SaPIs), and the mobilizable transposon of *Streptococcus agalactiae* MTnSag1, whose mobility depends on Tn*916*.^5,30-36^ The identification of *oriT*_SXT_ and elucidation of the master activator SetCD of SXT/R391 ICEs also greatly helped our recent investigation on A/C-dependent MGIs.^26,37^ Indeed, localization of *oriT*_SXT_ allowed the identification of similar chromosomal loci.^38^ Further investigations revealed that these chromosomal *oriT* sequences belonged to integrated MGIs, whose mobilization mechanism is slightly different from the above-described MGIs (Fig. 1C).^12,28,37-39^ Excision of these elements depends on the SetCD-dependent transcriptional activation of the RDF gene.^12,28,37^ *oriT*_MGI_ mimics *oriT*_SXT_; hence it is recognized by MobI_SXT_ and processed by the ICE-encoded relaxosome prior to transfer through the ICE-encoded mating pore (Fig. 1C). In the recipient cell, the MGI integrates autonomously due to *int* constitutive expression.^12,28^

## Concluding remarks

The propensity of MGIs to persist into and disseminate between bacterial populations using diverse strategies indicates that they are not defective elements. MGIs have rather adapted to act as parasites of self-transmissible MGEs, while at the same time spreading adaptive traits such as resistance to multiple antimicrobial compounds. An interesting example of this cooperative/antagonistic relationship is provided by SGI1 and variants that rely on IncC plasmids for mobilization.^16^ Co-transfer of plasmid and GI is rare suggesting that the latter is able to affect plasmid transfer, as also confirmed by the rapid loss of the plasmid when SGI1 is co-present in the same *E. coli* cell.^40^

This captivating research area is likely to deepen our understanding of other families of MGEs, including other classes of MGIs. Future investigations must focus on (i) the characterization of master regulators and cognate target sequences of a broad set of self-transmissible MGEs and (ii) the identification of their *oriT* sequence. Such valuable information will help when performing data mining of genome sequences to identify new DNA elements acquired by horizontal gene transfer. In-depth, step-by-step discoveries will help paving the road to building an atlas of interconnections between MGEs and associated massive flow of genetic material.

## Abbreviations

A/C: plasmids of incompatibility groups A and C
GI: genomic island
ICE: integrative conjugative element
MGE: mobile genetic element
MGI: mobilizable genomic island
*oriT*: origin of transfer
